# Supplementary material for: Mathematical Modeling Suggests Cooperation of Plant-Infecting Viruses
Source: Viruses. 2022 Mar 31;14(4):741. doi: 10.3390/v14040741 (PMC9029262; doi:10.3390/v14040741)
Supplement: Supplementary file 1 [file viruses-14-00741-s001.zip › viruses-1608454-supplementary.pdf]

Supplemental Information

Mathematical modeling suggests cooperation of plant-infecting viruses

Joshua Miller, Tessa Burch-Smith, and Vitaly V. Ganusov

Additional figures and tables

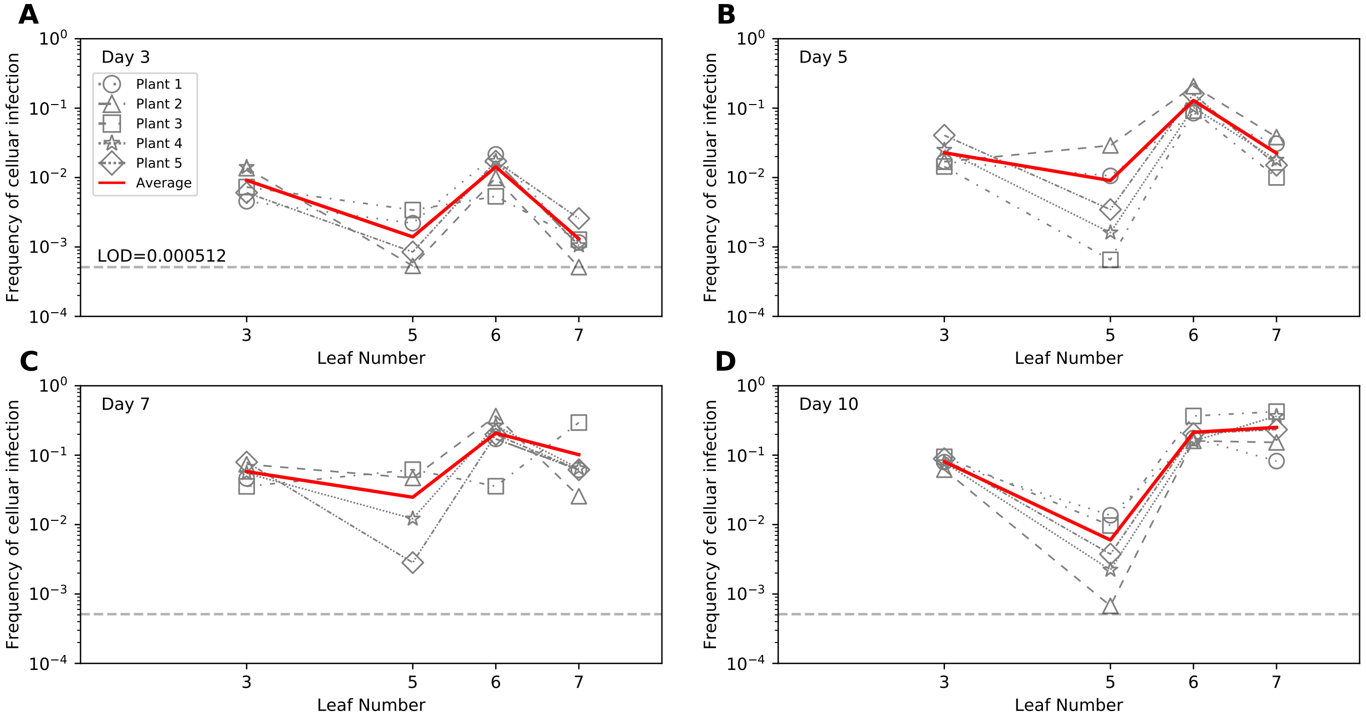

Supplemental Figure S1: Kinetics of TEV dissemination in tobacco plants partitioned per individual plant show variability in leaf infection levels. We plot the data on infection of cells with either or both variants of TEV for individual leaves of a given plant for 3 (A), 5 (B), 7 (C), or 10 (D) days since infection. Symbols denote the frequency of infected cells in a leaf with lines connecting measurements in individual plants. Solid red line denotes average infection per leaf for a given time point.

Fitted with binomial distribution-based likelihood method								
Parameter	Original	Alt. Model 1	Alt. Model 2	Alt. Model 3	Alt. Model 4	Alt. Model 5	Alt. Model 6	Alt. Model 7
I_0	.0003	.0005	.0004	.00006	.0001	.0006	.0001	.0001
β, 1/day	.950	.837	.887	1.289	1.040	1.000	1.037	1.032
χ_3, 1/day	N/A	N/A	N/A	N/A	.391	.042	.385	.064
χ_5, 1/day	.167	.120	.135	.775	N/A	1.876	.239	.047
χ_6, 1/day	1.046	5.489	4.964	8.101	2.275	N/A	2.269	2.251
χ_7, 1/day	.029	.059	.465	.972	.193	.022	N/A	N/A
ψ_3	.080	.083	.081	.075	.074	.075	.072	.072
ψ_5	.016	.017	.016	.016	.016	.011	.016	.016
ψ_6	.224	.223	.223	.217	.225	.235	.219	.219
ψ_7	.269	.276	.276	.590	.265	.268	.269	.269
nll	378317	378212	378172	378343	378495	379377	378442	378524
AIC_{Lik}	756652	756442	756362	756704	757008	758772	756902	757066
Δ_{AIC}	290	80	0	342	646	2410	540	704

Supplemental Table S1: Alternative models for viral dissemination fitted to data using binomial distribution-based likelihood describe the data with different quality based on AIC values. We performed the same analysis as **Table 2** except that models were fitted to data using binomial distribution-based likelihood.

Param	I_0	β, 1/day	χ_5, 1/day	χ_6, 1/day	χ_7, 1/day						SSR_{Log}	$AIC_{SSR_{Log}}$	Δ_{AIC}
Fixed n						T_3	T_5	T_6	T_7	n			
	0.0005	0.829	0.061	0.724	0.032	5.762	4.194	5.192	7.654	7.122	52.960	-13	3
Fixed T						n_3	n_5	n_6	n_7	T			
	0.00008	2.518	0.049	0.835	0.055	3.524	6.017	3.659	1.82E+00	3.2	53.073	-13	3
Fixed n, T						T	n						
	0.001	0.673	0.025	0.602	0.068	6.127	9.316				67.555	0	16

Supplemental Table S2: A mathematical model assuming that virus dissemination is influenced by the leaf-specific and systemic immunity can describe the experimental data. We changed the original, Tromas *et al.* [14] model by assuming the time-dependent and leaf-dependent S_k function (Alternative model 9, see eqn. (17)) and fitted the model to the data using least squares with a logarithmic transform eqn. (35). In fits we either varied the time (T_k) at which S_k declines to zero, the Hill coefficient (n_k) which determines the speed at which S_k declines to zero, or both parameters being independent of the leaf number (k). The resulting SSR_{Log} and $AIC_{SSR_{Log}}$ values for different model fits are shown (AICs are rounded to the nearest whole number). We found the following values of the alternative model 8 (eqn. (16)) fits of the data: $SSR_{Log} = 52.285$ and $AIC_{SSR_{Log}} = -10$.

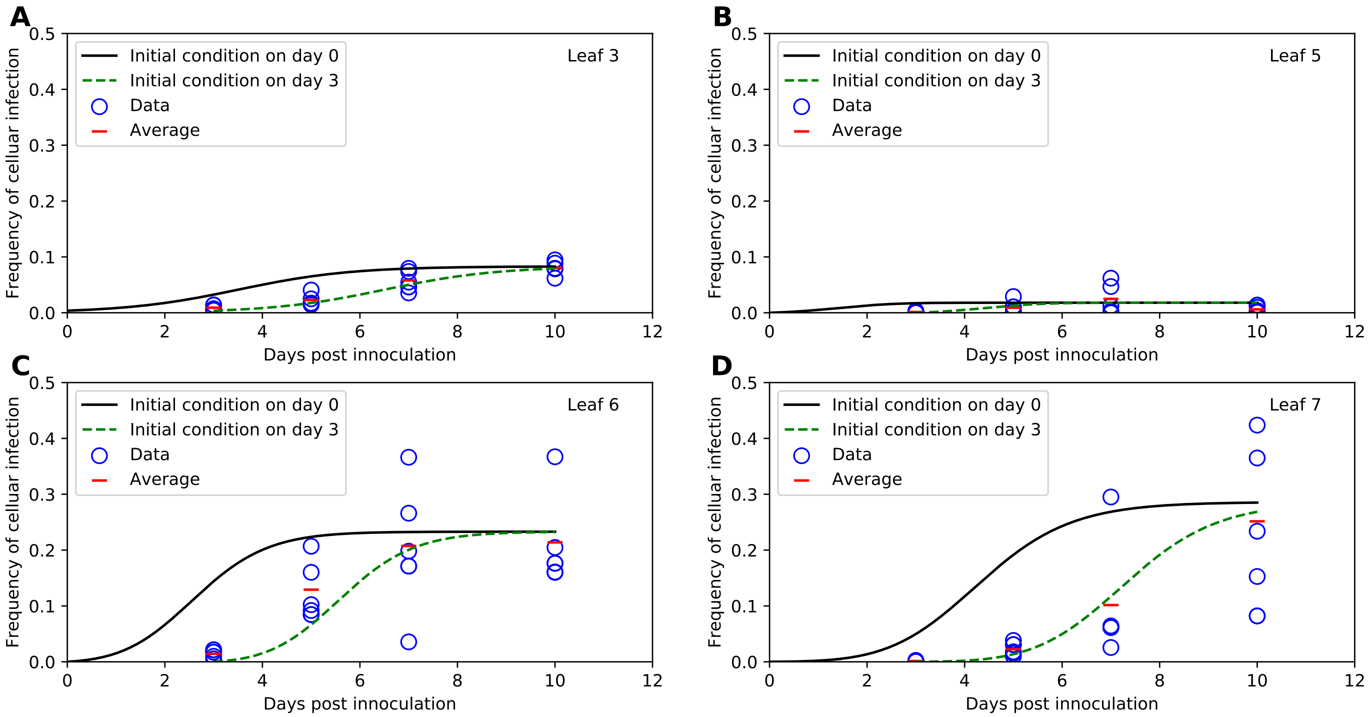

Supplemental Figure S2: Shifting original model predictions (found in Tromas *et al.* [14]) by three days reasonably well matches experimental data. We integrated the original model (given in eqns. (1)–(3)) using an ODE solver in python either assuming that infection starts at day 0 (solid lines) or infection starts at day 3 (dashed lines); data are shown by markers for leaf 3 (A), leaf 5 (B), leaf 6 (C), and leaf 7 (D). By default, ODE solver in python is initialized by the first time point provided in the data which is day 3 in the data set. We overrode the default by forcing the solver to start infection at day 0.

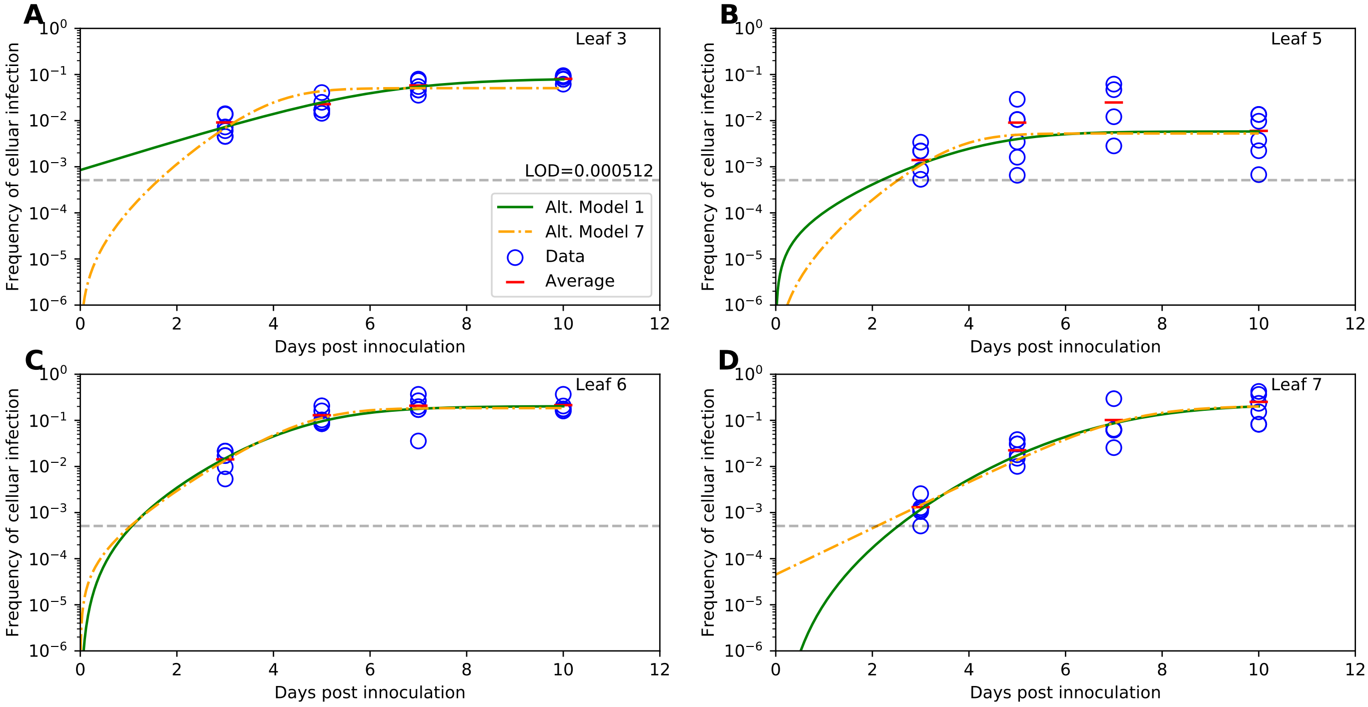

Supplemental Figure S3: The difference between the best performing alternative model 1 (eqn. (4)) and the worst-performing, alternative model 7 (eqn. (15)) is visually small. We fitted the two models using the least-squares method with a log transformation where zeros were replaced with a limit of detection model (LOD) value, in this case $5.12 \cdot 10^{-4}$. The parameters for Alt Model 1 with 95% confidence intervals are: $I_0 = 0.0008\ (0.0002, 0.0017)$, $\beta = 0.744\ (0.554, 1.308)$, $\chi_5 = 0.059\ (0.033, 0.119)$, $\chi_6 = 8.201\ (4.151, 13.206)$, $\chi_7 = 0.073\ (0.052, 0.152)$, $\psi_3 = 0.083\ (0.059, 0.117)$, $\psi_5 = 0.006\ (0.005, 0.013)$, $\psi_6 = 0.204\ (0.186, 0.227)$, $\psi_7 = 0.228\ (0.081, 0.699)$. The parameters for Alt. Model 7 with 95% confidence intervals are: $I_0 = 0.0005\ (2 \cdot 10^{-6}, 1 \cdot 10^{-5})$, $\beta = 1.159\ (0.973, 1.898)$, $\chi_3 = 0.286\ (0.117, 0.645)$, $\chi_5 = 0.050\ (0.023, 20)$, $\chi_6 = 3.263\ (2.365, 5.890)$, $\psi_3 = 0.051\ (0.041, 0.069)$, $\psi_5 = 0.005\ (0.003, 0.008)$, $\psi_6 = 0.184\ (0.143, 0.226)$, $\psi_7 = 0.209\ (0.081, 0.415)$. The goodness of fit metrics for Alt. Model 1 are: $SSR_{Log} = 51.991$ and $AIC_{SSR_{Log}} = -16$, and for comparison the Alt. Model 7's metrics are: $SSR_{Log} = 56.309$ and $AIC_{SSR_{Log}} = -10$, giving $\Delta_{AIC} = 6$.

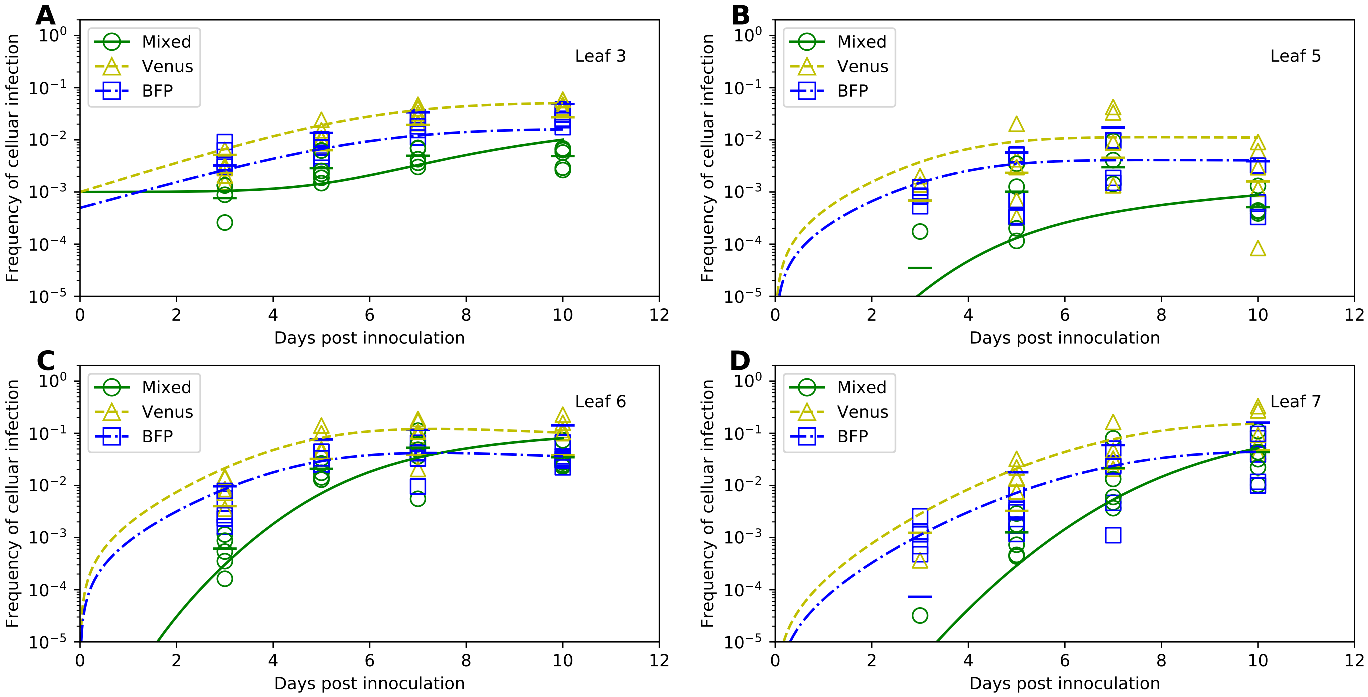

Supplemental Figure S4: The simplest 1-alpha coinfection model does not adequately describe coinfection data. The 1-alpha coinfection model (given by eqns. (18)–(19) and eqn. (21)) assumes that coinfection of individual cells by two different strains occurs independently ($\alpha = 1$) or coinfection may be more ($\alpha > 1$) or less ($0 < \alpha < 1$) likely that infection of an uninfected cell (**Figure 2**A). Other graph details are similar to those given in **Figure 6**. The parameters and 95% confidence intervals for this model are: $V_0 = .0006\ (.0003, .001)$, $B_0 = .0003\ (0.3 \times 10^{-5}, .001)$, $M_0 = .0001\ (.0004, .001)$, $\beta_V = .744\ (.443, 6.178)$/day, $\beta_B = .666\ (.260, 6.580)$/day, $\chi_5 = .269\ (.034, 8.76)$/day, $\chi_6 = .939\ (.395, 2.292)$/day, $\chi_7 = .044\ (.015, 2.107)$/day, $\psi_3 = .078\ (.044, .954)$, $\psi_5 = .016\ (.006, .028)$, $\psi_6 = .201\ (.156, .267)$, $\psi_7 = .254\ (.100, .449)$, $\alpha = 2.332\ (.103, 5.772)$.

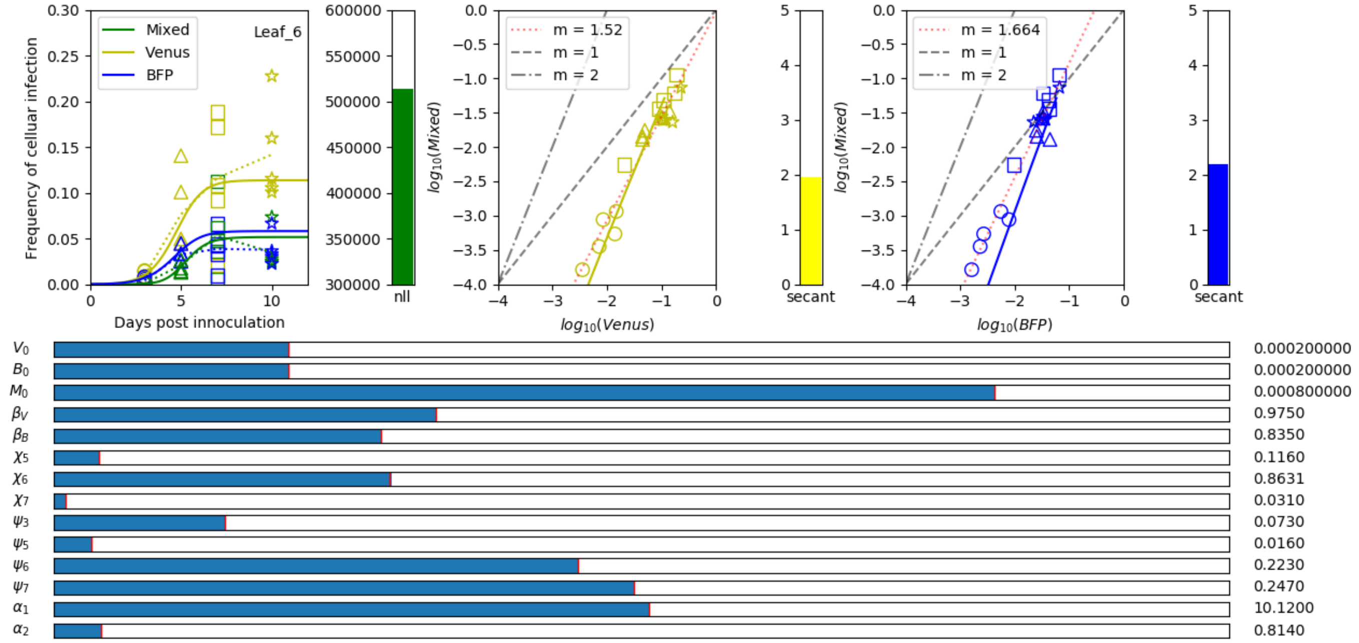

Supplemental Figure S5: A python-based tool to study the impact of parameters of a given mathematical model on the infection rate of a given leaf. By using a function `Slider` from `pylab` in python we visualized the dynamics of cell infection by individual viruses (Venus and BFP) and coinfection of cells by the two viruses according to the Probabilistic Analytic Model. Parameters of the model can be changed using sliders resulting in the changed kinetics of virus infection (shown in the left panel), or changes in the predicted relationships between the degree of coinfection of cells by two viruses (denoted as “Mixed”) and singly infected cells (Venus or BFP for middle and right panels, respectively). The example shown is for infection of leaf 6; the code allows to chose any individual leaf for visualization. In all panels data are shown by markers and predictions of the model by lines. Additional parameters shown are i) the negative log-likelihood (nll, see eqns. (30)–(33)); ii) the average ratio of the frequency of coinfected cells to singly-infected cells (secant); iii) the values of the expressions $m_{\frac{M}{V}}$ and $m_{\frac{M}{B}}$ evaluated at the rightmost timepoint in the leftmost panel, in this case t=12 ($m_{\frac{M}{V}}$ and $m_{\frac{M}{B}}$).

Expansion of the Tromas *et al.* [14] model

$$\frac{dI_3}{dt} = \beta I_3 S_3, \tag{S1}$$
$$\frac{dI_5}{dt} = \beta I_5 S_5 + \chi_5 S_5 I_3, \tag{S2}$$
$$\frac{dI_6}{dt} = \beta I_6 S_6 + \chi_6 S_6 (I_3 + I_5), \tag{S3}$$
$$\frac{dI_7}{dt} = \beta I_7 S_7 + \chi_7 S_7 (I_3 + I_5 + I_6). \tag{S4}$$

Alternative formulations of coinfection models

Expansion of the 1-alpha coinfection model (eqns. (18)–(20) and eqn. (21)):

$$\frac{dV_3}{dt} = \beta_V V_3 S_3, \tag{S5}$$
$$\frac{dB_3}{dt} = \beta_B B_3 S_3, \tag{S6}$$
$$\frac{dM_3}{dt} = \alpha(\beta_B V_3 B_3 + \beta_V V_3 B_3), \tag{S7}$$
$$\frac{dV_5}{dt} = \beta_V V_5 S_5 + \chi_5 S_5 V_3, \tag{S8}$$
$$\frac{dB_5}{dt} = \beta_B B_5 S_5 + \chi_5 S_5 B_3, \tag{S9}$$
$$\frac{dM_5}{dt} = \alpha(\beta_B V_5 B_5 + \beta_V V_5 B_5), \tag{S10}$$
$$\frac{dV_6}{dt} = \beta_V V_6 S_6 + \chi_6 S_6 (V_3 + V_5), \tag{S11}$$
$$\frac{dB_6}{dt} = \beta_B B_6 S_6 + \chi_6 S_6 (B_3 + V_5), \tag{S12}$$
$$\frac{dM_6}{dt} = \alpha(\beta_B V_6 B_6 + \beta_V V_6 B_6), \tag{S13}$$
$$\frac{dV_7}{dt} = \beta_V V_7 S_7 + \chi_7 S_7 (V_3 + V_5 + V_6), \tag{S14}$$
$$\frac{dB_7}{dt} = \beta_B B_7 S_7 + \chi_7 S_7 (B_3 + B_5 + B_6), \tag{S15}$$
$$\frac{dM_7}{dt} = \alpha(\beta_B V_7 B_7 + \beta_V V_7 B_7). \tag{S16}$$

Expansion of the 2-alpha coinfection model (eqns. (18)–(20) and eqn. (22)):

$$\frac{dV_3}{dt} = \beta_V V_3 S_3, \tag{S17}$$
$$\frac{dB_3}{dt} = \beta_B B_3 S_3, \tag{S18}$$
$$\frac{dM_3}{dt} = \alpha_B \beta_B V_3 B_3 + \alpha_V \beta_V V_3 B_3, \tag{S19}$$
$$\frac{dV_5}{dt} = \beta_V V_5 S_5 + \chi_5 S_5 V_3, \tag{S20}$$
$$\frac{dB_5}{dt} = \beta_B B_5 S_5 + \chi_5 S_5 B_3, \tag{S21}$$
$$\frac{dM_5}{dt} = \alpha_B \beta_B V_5 B_5 + \alpha_V \beta_V V_5 B_5, \tag{S22}$$
$$\frac{dV_6}{dt} = \beta_V V_6 S_6 + \chi_6 S_6 (V_3 + V_5), \tag{S23}$$
$$\frac{dB_6}{dt} = \beta_B B_6 S_6 + \chi_6 S_6 (B_3 + V_5), \tag{S24}$$
$$\frac{dM_6}{dt} = \alpha_B \beta_B V_6 B_6 + \alpha_V \beta_V V_6 B_6, \tag{S25}$$
$$\frac{dV_7}{dt} = \beta_V V_7 S_7 + \chi_7 S_7 (V_3 + V_5 + V_6), \tag{S26}$$
$$\frac{dB_7}{dt} = \beta_B B_7 S_7 + \chi_7 S_7 (B_3 + B_5 + B_6), \tag{S27}$$
$$\frac{dM_7}{dt} = \alpha_B \beta_B V_7 B_7 + \alpha_V \beta_V V_7 B_7. \tag{S28}$$

Expansion of the 1-alpha probabilistic model (eqns. (18)–(20) and eqn. (24)):

$$\frac{dV_3}{dt} = \beta_V V_3 S_3, \tag{S29}$$
$$\frac{dB_3}{dt} = \beta_B B_3 S_3, \tag{S30}$$
$$\frac{dM_3}{dt} = \alpha[S_3 V_3 B_3(\beta_B + \beta_V)], \tag{S31}$$
$$\frac{dV_5}{dt} = \beta_V V_5 S_5 + \chi_5 S_5 V_3, \tag{S32}$$
$$\frac{dB_5}{dt} = \beta_B B_5 S_5 + \chi_5 S_5 B_3, \tag{S33}$$
$$\frac{dM_5}{dt} = \alpha[S_5 B_5 V_5(\beta_B + \beta_V) + \chi_5 S_5(B_5 V_3 + V_5 B_3)], \tag{S34}$$
$$\frac{dV_6}{dt} = \beta_V V_6 S_6 + \chi_6 S_6(V_3 + V_5), \tag{S35}$$
$$\frac{dB_6}{dt} = \beta_B B_6 S_6 + \chi_6 S_6(B_3 + B_5), \tag{S36}$$
$$\frac{dM_6}{dt} = \alpha[(S_6 V_6 B_6(\beta_V + \beta_B) + \chi_6 S_6(B_6(V_3 + V_5) + V_6(B_3 + B_5))], \tag{S37}$$
$$\frac{dV_7}{dt} = \beta_V V_7 S_7 + \chi_7 S_7(V_3 + V_5 + V_6), \tag{S38}$$
$$\frac{dB_7}{dt} = \beta_B B_7 S_7 + \chi_7 S_7(B_3 + B_5 + B_6), \tag{S39}$$
$$\frac{dM_7}{dt} = \alpha[(S_7 V_7 B_7(\beta_V + \beta_B) + \chi_7 S_7(B_7(V_3 + V_5 + V_6) + V_7(B_3 + B_5 + B_6))]. \tag{S40}$$

Deriving relationship between coinfected and single-infected cells

We found that the relationship between the frequency of coinfected cells and of singly infected cells is approximately linear (e.g., **Figure 7**). To understand this we performed the following analyses. Specifically, we aim at calculating asymptotic behavior of $\frac{dM_k}{dV_k}$ and $\frac{dM_k}{dB_k}$.

Derivation of the "V_k" case

Using basic calculus and eqn. (18), eqn. (19), and eqn. (23) we find:

$$\frac{dM_k}{dV_k} = \frac{\frac{dM_k}{dt}}{\frac{dV_k}{dt}} = \frac{\alpha[B_k V_k' + V_k B_k']}{V_k'} = \alpha\left[B_k + \frac{V_k B_k'}{V_k'}\right]. \tag{S41}$$

where $'$ denotes derivative in time. The key to the behavior of the relationship between coinfected and single-infected cells thus lies in understanding the behavior of

$$\frac{V_k B_k'}{V_k'}. \tag{S42}$$

Leaf 3. We first consider the leaf 3 as it is the simplest and provides a method we can use to understand patterns for higher leaves. Simplifying eqn. (S42) gives:

$$\frac{V_3 B_3'}{V_3'} = \frac{V_3(\beta_B S_3 B_3)}{\beta_V S_3 V_3} = \frac{\beta_B}{\beta_V} B_3. \tag{S43}$$

Using this, we can find the expression for the original equation.

$$\frac{dM_k}{dV_k} = \alpha \left[B_3 + \frac{\beta_B}{\beta_V} B_3 \right] = \alpha \left(1 + \frac{\beta_B}{\beta_V} \right) B_3 = c B_3. \tag{S44}$$

Further leaves. In the cases where $k > 3$ we have:

$$\frac{V_k B_k'}{V_k'} = \frac{V_k(\beta_B S_k B_k + \chi_k S_k \sum_{i=3}^{k-1} B_i)}{\beta_V S_k V_k + \chi_k S_k \sum_{i=3}^{k-1} V_i}. \tag{S45}$$

This expression is much more difficult to simply than the $k = 3$ case. However, if we take a linear combination between V_3, V_5, etc. we can proceed. For simplicity, we can use the average:

$$\overline{V} = \frac{1}{n+1} \left(V_k + \sum_{i=3}^{k-1} V_i \right), \qquad \overline{B} = \frac{1}{n+1} \left(B_k + \sum_{i=3}^{k-1} B_i \right), \tag{S46}$$

where n is the number of proper leaves below the kth leaf. Using this eqn. (S45) becomes:

$$\frac{\overline{V}(\beta_B S_k \overline{B} + \chi_k S_k \overline{B})}{\beta_V S_k \overline{V} + \chi_k S_k \overline{V}} = \overline{B} \frac{\beta_B + \chi_k}{\beta_V + \chi_k}. \tag{S47}$$

And thus we have:

$$\frac{dM_k}{dV_k} = \alpha \left[\overline{B} + \overline{B} \frac{\beta_B + \chi_k}{\beta_V + \chi_k} \right] = \alpha \left(1 + \frac{\beta_B + \chi_k}{\beta_V + \chi_k} \right) \overline{B} = c \overline{B}. \tag{S48}$$

Because $\overline{V}$ and $\overline{B}$ are linear functions of V_k and B_k, we can conclude that indeed $\frac{dM_k}{dV_k}$ is proportional to B_k, and by inference, $\frac{dM_k}{dB_k}$ is proportional to V_k.

Derivation of the "B_k" case

Proceeding similarly as with eqn. (S41) we find

$$\frac{\frac{dM_k}{dt}}{\frac{dB_k}{dt}} = \frac{\alpha[B_k V_k' + V_k B_k']}{B_k'} = \alpha\left[\frac{B_k V_k'}{B_k'} + V_k\right]. \tag{S49}$$

Leaf 3.

$$\frac{B_3 V_3'}{B_3'} = \frac{B_3(\beta_V S_3 V_3)}{\beta_B S_3 B_3} = \frac{\beta_V}{\beta_B} V_3 \implies \frac{\frac{dM_k}{dt}}{\frac{dV_k}{dt}} = \alpha\left[\frac{\beta_V}{\beta_B} V_3 + V_3\right] = \alpha\left(\frac{\beta_V}{\beta_B} + 1\right) V_3 = cV_3. \tag{S50}$$

Further leaves. In the cases where $k > 3$ we have:

$$\frac{B_k V_k'}{B_k'} = \frac{V_k(\beta_V S_k V_k + \chi_k S_k \sum_{i=3}^{k-1} V_i)}{\beta_B S_k B_k + \chi_k S_k \sum_{i=3}^{k-1} B_i}. \tag{S51}$$

Let

$$\overline{V} = \frac{1}{n+1}\left(V_k + \sum_{i=3}^{k-1} V_i\right), \qquad \overline{B} = \frac{1}{n+1}\left(B_k + \sum_{i=3}^{k-1} B_i\right), \tag{S52}$$

where n is the number of proper leaves below the kth leaf.

$$\frac{B_k V_k'}{B_k'} \to \frac{\overline{B}(\beta_V S_k \overline{V} + \chi_k S_k \overline{V})}{\beta_B S_k \overline{B} + \chi_k S_k \overline{B}} = \overline{V}\frac{\beta_V + \chi_k}{\beta_B + \chi_k}. \tag{S53}$$

And thus we have:

$$\frac{dM_k}{dB_k} = \alpha\left[\overline{V}\frac{\beta_V + \chi_k}{\beta_B + \chi_k} + \overline{V}\right] = \alpha\left(\frac{\beta_B + \chi_k}{\beta_V + \chi_k} + 1\right)\overline{V} = c\overline{V}. \tag{S54}$$
